# Supplementary material for: Spatial suppression promotes rapid figure-ground segmentation of moving objects
Source: Nat Commun. 2019 Jul 2;10:2732. doi: 10.1038/s41467-019-10653-8 (PMC6606582; doi:10.1038/s41467-019-10653-8)
Supplement: Supplementary file 2 — Reporting Summary [file 41467_2019_10653_MOESM2_ESM.pdf]

natureresearch

Corresponding author(s): DUJE TADIN

Last updated by author(s): May 14, 2019

Reporting Summary

Nature Research wishes to improve the reproducibility of the work that we publish. This form provides structure for consistency and transparency in reporting. For further information on Nature Research policies, see Authors & Referees and the Editorial Policy Checklist.

Statistics

For all statistical analyses, confirm that the following items are present in the figure legend, table legend, main text, or Methods section.

n/a	Confirmed	
☐	☒	The exact sample size (*n*) for each experimental group/condition, given as a discrete number and unit of measurement
☒	☐	A statement on whether measurements were taken from distinct samples or whether the same sample was measured repeatedly
☐	☒	The statistical test(s) used AND whether they are one- or two-sided<br>*Only common tests should be described solely by name; describe more complex techniques in the Methods section.*
☒	☐	A description of all covariates tested
☐	☒	A description of any assumptions or corrections, such as tests of normality and adjustment for multiple comparisons
☐	☒	A full description of the statistical parameters including central tendency (e.g. means) or other basic estimates (e.g. regression coefficient) AND variation (e.g. standard deviation) or associated estimates of uncertainty (e.g. confidence intervals)
☐	☒	For null hypothesis testing, the test statistic (e.g. *F*, *t*, *r*) with confidence intervals, effect sizes, degrees of freedom and *P* value noted<br>*Give P values as exact values whenever suitable.*
☒	☐	For Bayesian analysis, information on the choice of priors and Markov chain Monte Carlo settings
☒	☐	For hierarchical and complex designs, identification of the appropriate level for tests and full reporting of outcomes
☐	☒	Estimates of effect sizes (e.g. Cohen's *d*, Pearson's *r*), indicating how they were calculated

Our web collection on statistics for biologists contains articles on many of the points above.

Software and code

Policy information about availability of computer code

Data collection	Custom code in Matlab and Psychophysics Toolbox was used to collect data. This is standard in psychophysical vision research.
Data analysis	Matlab and SPSS were used for all standard data analysis (ANOVA, correlations). Custom Matlab code was created for the model fitting and bootstrap analysis. All custom code used in the project is available upon request.

For manuscripts utilizing custom algorithms or software that are central to the research but not yet described in published literature, software must be made available to editors/reviewers. We strongly encourage code deposition in a community repository (e.g. GitHub). See the Nature Research guidelines for submitting code & software for further information.

Data

Policy information about availability of data

All manuscripts must include a data availability statement. This statement should provide the following information, where applicable:

- Accession codes, unique identifiers, or web links for publicly available datasets
- A list of figures that have associated raw data
- A description of any restrictions on data availability

The data sets generated during and/or analyzed during the current study (Fig. 1-7) are available from the corresponding author on request. Modeling computer code (MATLAB) is also available on request.

Field-specific reporting

Please select the one below that is the best fit for your research. If you are not sure, read the appropriate sections before making your selection.

☐ Life sciences ☒ Behavioural & social sciences ☐ Ecological, evolutionary & environmental sciences

For a reference copy of the document with all sections, see nature.com/documents/nr-reporting-summary-flat.pdf

Behavioural & social sciences study design

All studies must disclose on these points even when the disclosure is negative.

Study description	Quantitative psychophysical study of visual perception.
Research sample	The sample consists of Vanderbilt University and University of Rochester students and staff, as well as high-functioning community dwelling older adults in Rochester NY. Age varied from 18 to 84, with a balance of male and female subjects. This study sample allowed us to balance representativeness and convenience of recruitment.
Sampling strategy	For experiments not involving individual differences, we aimed for samples that matched original work on this topic (Tadin et al., 2003, Nature) which tested 3-5 participants per experiment (we tested 4-6). For individual differences work, we aimed for samples that are about an order of magnitude larger.
Data collection	All data was collected with a computer using button presses, with an experimenter not in the same room at the participant (instead the experimenter waited in the adjacent room). No blinding of the experimenter was used.
Timing	This study is a combination of several experiments conducted over a longer period of time. The oldest experiment was conducted (i.e., data collected) in 2003, while the data for the most recent one was collected in the fall of 2014. Although the whole study spanned a large period of time, data for individual experiments were collected over much shorter periods of time (on the order of months).
Data exclusions	Data for 1 young and 3 older adults were excluded because data variability (defined as standard deviation, SD, of threshold measurements for individual conditions) was 3 SD higher than the average variability
Non-participation	No participants dropped out. We did not keep records on declined participation, but such declines occur very rarely in our studies.
Randomization	These was no allocations of participants into experimental groups.

Reporting for specific materials, systems and methods

We require information from authors about some types of materials, experimental systems and methods used in many studies. Here, indicate whether each material, system or method listed is relevant to your study. If you are not sure if a list item applies to your research, read the appropriate section before selecting a response.

Materials & experimental systems

n/a	Involved in the study	
☒	☐	Antibodies
☒	☐	Eukaryotic cell lines
☒	☐	Palaeontology
☒	☐	Animals and other organisms
☐	☒	Human research participants
☒	☐	Clinical data

Methods

n/a	Involved in the study	
☒	☐	ChIP-seq
☒	☐	Flow cytometry
☒	☐	MRI-based neuroimaging

Human research participants

Policy information about studies involving human research participants

Population characteristics	See above
Recruitment	Majority of subjects were recruited using recruitment flyers. For older adults, we placed flyers in community centers and older adult education places. For low level vision, we do not expect any issues with self-selection bias.
Ethics oversight	institutional review boards at Vanderbilt University and University of Rochester

Note that full information on the approval of the study protocol must also be provided in the manuscript.
